# Reassessing the role of CEA in the treatment of rectal cancer in the era of TNT

**DOI:** 10.1016/j.esmogo.2026.100355

**Published:** 2026-06-29

**Authors:** M. Diefenhardt, R.-D. Hofheinz, M. Fleischmann, A. Schlenska-Lange, R. Grützmann, S. Kirste, T. Kuhnt, R. Kosmala, C. Rödel, A. Wiegering, M. Ghadimi, C. Gani, E. Fokas, D. Martin

**Affiliations:** 1 Department of Radiotherapy, Goethe-University Frankfurt, University Hospital, Frankfurt, Germany; 2 Frankfurt Cancer Institute, Goethe-University Frankfurt, University Hospital, Frankfurt, Germany; 3 Department of Hematology and Oncology, University Medical Center Mannheim, Medical Faculty Mannheim, Heidelberg University, Mannheim, Germany; 4 Department of Hematology and Medical Oncology, Hospital Barmherzige Brüder Regensburg, Regensburg, Germany; 5 Department of General and Visceral Surgery, University of Erlangen-Nürnberg, Erlangen, Germany; 6 Department of Radiation Oncology, Medical Center – University of Freiburg, Faculty of Medicine, Freiburg, Germany; 7 Department of Radiation Oncology, University Hospital Leipzig, Leipzig, Germany; 8 Department of Radiotherapy and Radiation Oncology, University Hospital Würzburg, Würzburg, Germany; 9 German Cancer Research Center (DKFZ), Heidelberg, German Cancer Consortium (DKTK), Partner Site Frankfurt am Main, Frankfurt, Germany; 10 Department of General, Visceral, Transplantation, and Thoracic Surgery, Goethe-University Frankfurt, University Hospital, Frankfurt, Germany; 11 Department of General, Visceral and Pediatric Surgery, University Göttingen, University Hospital, Göttingen, Germany; 12 Department of Radiation Oncology, University Hospital Tübingen, Tübingen, Germany; 13 German Cancer Research Center (DKFZ), Heidelberg, German Cancer Consortium (DKTK), Partner Site Tübingen, Frankfurt, Germany; 14 Department of Radiation Oncology, Cyberknife and Radiation Therapy, University of Cologne, Faculty of Medicine and University Hospital Cologne, Cologne, Germany

**Keywords:** CEA, clinical trial, rectal cancer, surveillance, total neoadjuvant therapy

## Abstract

**Background:**

Measurement of carcinoembryonic antigen (CEA) is an integral part of surveillance strategies in rectal cancer. We evaluated the prognostic significance of CEA level before total neoadjuvant therapy (TNT) and its role in surveillance strategies after TNT.

**Patients and methods:**

This *post hoc* analysis of the CAO/ARO/AIO-12 trial involved 306 patients who were treated with induction chemotherapy (CT) followed by chemoradiotherapy (CRT), or CRT followed by CT, and then underwent total mesorectal excision.

**Results:**

Baseline CEA levels were available for 282 patients (92%). The median baseline CEA level was 5.45 ng/ml for patients with ypN+, 3.35 ng/ml for patients with ypT+, and 2.45 ng/ml for patients with complete response (CR: pathological CR + clinical CR). Only baseline CEA levels and baseline white blood cell count remained significant parameters in a best-fit generalized linear regression model predicting CR (area under the curve 0.725). In a multivariate Fine–Gray model, CEA was significantly associated with treatment failure (TF); TNT only showed a significant reduction in the risk of TF in the subgroup of patients with baseline CEA >10 ng/ml compared with patients treated with 5-fluorouracil (5-FU) CRT and adjuvant 5-FU in the CAO/ARO/AIO-04 trial. The risk of TF was 13% in patients with no CEA elevation >3 ng/ml during follow-up, compared with 54% (63%) in patients with CEA elevation >5 ng/ml (10 ng/ml). No local recurrence after TNT was associated with an elevated CEA level >5 ng/ml.

**Conclusions:**

Baseline CEA levels should be considered an integral part of risk-adapted treatment strategies and remain an essential and cost-effective part of surveillance strategies after TNT.

## Highlights

• Evaluating the value of CEA measurements in patients treated with TNT in a cohort from a prospective clinical phase II trial.
• Baseline CEA levels are associated with the probability of achieving a CR.
• CEA measurements remain a cost-effective component of surveillance strategies for detecting metastasis.
• ‘CEA-turns’ in patients with normal baseline CEA levels can occur and indicate metastasis during surveillance.
• No isolated local recurrence was associated with a meaningful CEA increase, indicating limit benefit in W&W approaches.

## Introduction

Multimodal treatment strategies for locally advanced rectal cancer have evolved dynamically in recent years, with a decrease in distant metastasis rates, improved survival, and development of organ-preserving approaches in selected patients.^1^, ^2^, ^3^ Despite this progress, one in four patients with locally advanced rectal cancer experiences treatment failure (TF), which is associated with a negative impact on overall survival (OS) and with deterioration in quality of life, even though the prognosis with salvage treatment has improved in recent years.^4^

The surveillance strategies recommended by international guidelines consist of clinical examination, radiological procedures, and measurement of carcinoembryonic antigen (CEA). However, recommendations for intensified surveillance protocols after nonresponse or limited response to neoadjuvant treatment (e.g. ypT4, ypN+) are lacking, even though these patients are at higher risk of TF.^5^^,^^6^

CEA proteins are cellular components that are suppressed during the differentiation of normal digestive system epithelium during fetal development. In 1964-1965, Gold and Freedman^7^^,^^8^ discovered that these antigens were present in malignant tumors originating from the gastrointestinal tract but absent in tumors of other origin. They hypothesized that CEA could reappear in malignant cells through a process of ‘derepressive dedifferentiation’. However, despite their initial hypothesis, CEA elevation has also been found in breast cancer, prostate cancer, and non-small cell lung cancer.^9^

Nevertheless, its sensitivity in the diagnosis of rectal cancer is limited, as many patients have no significant CEA elevation at all (e.g. pretreatment CEA levels <5 ng/ml in 65% of patients in the PRODIGE23 trial^10^), even in cases of distant metastasis or local recurrence. It is therefore not recommended for primary diagnosis.^11^ CEA measurements are widely available and, unlike radiological diagnostics, are not limited by financial constraints within the health care system. Moreover, the role of CEA to improve identification of patients who might benefit from intensified neoadjuvant treatment protocols as total neoadjuvant therapy (TNT) and the value of CEA measurements in detection of recurrence in the era of selective organ preservation approaches is unclear. In this study, we report a *post hoc* analysis that addresses the potential role of CEA in 306 patients who were treated with two TNT sequences in the CAO/ARO/AIO-12 trial.

## Patient and methods

### Study design and participants

In the CAO/ARO/AIO-12 randomized phase II trial, patients with locally advanced rectal cancer were treated either with induction chemotherapy (CT) before chemoradiotherapy (CRT) or consolidation CT after CRT. Details of the inclusion and exclusion criteria, treatment procedures, and long-term outcomes have already been published.^12^^,^^13^ Even if surgery was mandatory, some patients refused surgery due to clinical complete response (cCR). Patients with a pathological complete response (pCR) or clinical complete response (cCR) were jointly classified as having a complete response (CR). To investigate the potential impact of CEA after an intensified neoadjuvant treatment protocol, we additionally examined data from 1236 patients who were treated in the CAO/ARO/AIO-04 trial. These patients were either treated with 5-fluorouracil (5-FU) CRT, total mesorectal excision (TME) surgery and adjuvant 5-FU CT, or with 5-FU–oxaliplatin CRT, TME surgery, and adjuvant 5-FU–oxaliplatin CT.^14^ In the CAO/ARO/AIO-12 trial, the CEA measurements were taken at screening, after induction CT or CRT, before surgery, and 4 weeks after surgery. Follow-up CEA measurements were taken every 3 months for the first 3 years, every 6 months in year 4 and once in year 5. In the CAO/ARO/AIO-04 trial, CEA measurements were taken at screening and during follow-up, in the same way as in the CAO/ARO/AIO-12 trial. The CAO/ARO/AIO-12 trial is registered at ClinicialTrials.gov (NCT02363374).

### Statistical analyses

The baseline CEA distribution, the CEA distribution after TNT, and the CEA distribution after TME surgery were plotted descriptively according to pathological outcome. We analyzed the baseline continuous CEA distribution and its correlation with clinical parameters using the Spearman correlation test, Mann–Whitney *U* test, or Kruskal–Wallis test.

To investigate the potential of CEA together with other baseline clinical and laboratory parameters to predict CR, we identified a best-fitting generalized linear regression model using the *bestglm* function in the *bestglm* package in R statistical software. Initially, the following parameters were included: baseline CEA levels, sex, Eastern Cooperative Oncology Group (ECOG) performance status, cT category, cN category (according to AJCC 7th), grading, age, body mass index (BMI), hemoglobin levels, leukocyte counts, creatinine levels, platelet counts, bilirubin levels, and alkaline phosphatase levels, as well as treatment arm. Internal model validation was carried out using the *aucadj* function to estimate the amount of overfitting and the *model.valid* function to perform and visualize the results of an internal validation by splitting the dataset randomly into a fitting (75%) and a validation (25%) portion.^15^

The association of baseline CEA levels with the risk of TF was assessed using Gray’s test and multivariate Fine–Gray regression. Finally, we investigated the benefit of TNT compared with neoadjuvant CRT in CEA-adjusted subgroup analyses using patient data from the CAO/ARO/AIO-04 trial. As CEA levels <3 ng/ml are generally considered normal for nonsmokers and ≤5 ng/ml normal for smokers and elevations ≤10 ng/ml as slightly and ≥10 ng/ml as highly elevated, we stratified our cohort for this analysis accordingly.^9^

Before this, violations of the proportional hazards assumption (using the *cox.zph* function) or multicollinearity (calculating the variance inflation factor) were ruled out. The reverse Kaplan–Meier approach was used to calculate the median follow-up time. *P* < 0.05 was considered statistically significant. Due to the exploratory nature of this analysis, no adjustment for multiple testing was made. All analyses were carried out using R statistical software version 4.5.

## Results

### Patient cohort

Between June 2015 and January 2018, 311 patients were enrolled in the CAO/ARO/AIO-12 trial, of whom 306 were eligible for this analysis. A total of 1265 patients were enrolled in the CAO/ARO/AIO-04 trial, of whom 1232 were eligible for the comparison analysis. The baseline patient demographics, clinical and pathological characteristics, and long-term oncological outcomes of both trials have been reported previously.^12^, ^13^, ^14^^,^^16^

### Baseline CEA distribution

**Figure 1:**
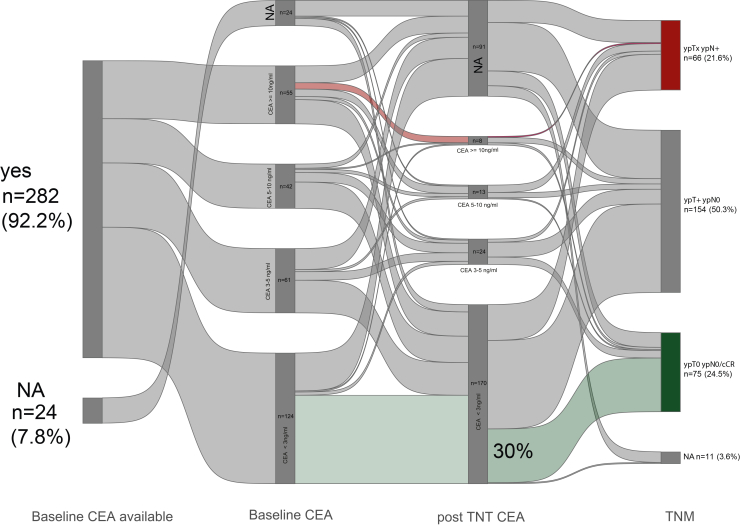
**Sankey diagram of CEA distribution at baseline, after TNT and its association with pathological outcome.** CEA, carcinoembryonic antigen.

Baseline CEA levels were available for 282 of 306 patients (92.2%; Figure 1). The median CEA level was 3.4 ng/ml (mean 9.5 ng/ml). There was no significant difference in CEA levels by any baseline clinical parameter (Table 1). Platelet counts and baseline alkaline phosphatase levels correlated significantly positively with CEA levels, whereas creatine and bilirubin levels correlated significantly negatively with CEA levels (Supplementary Table S1, available at https://doi.org/10.1016/j.esmogo.2026.100355).

**Table 1:** Correlation of clinical parameters with pretreatment CEA levels

Parameter	Number	Median CEA (ng/ml)	Mean CEA (ng/ml)	Standard deviation	*P*-value
All	282	3.4	9.5	19.4	
Sex					
Male	189	3.5	8.3	12.7	
Female	93	3.2	12.0	28.6	0.820^a^
Age (years)					
<60	136	4.0	10.1	18.4	
60-70	91	3.0	9.7	16.8	
≥70	55	3.0	7.9	25.4	0.154^b^
cT category					
2	10	4.8	6.4	7.7	
3	231	3.2	8.3	17.4	
4	41	4.6	17.1	2.8	0.088^b^
cN category					
0	24	2.8	9.9	19.2	
1	251	3.4	9.5	19.7	0.786^a^
ECOG					
0	203	3.1	9.2	20.3	
1	73	4.1	10.7	17.7	0.090^b^
Tumor localization					
Low	120	3.9	10.1	19.1	
Intermediate	133	3.6	10.3	21.4	
high	22	2.4	4.5	6.4	0.078^b^
Grading					
1	15	2.8	3.9	5.3	
2	225	3.4	10.2	21.0	
3	18	3.6	9.5	15.4	0.254^b^

CEA, carcinoembryonic antigen; ECOG, Eastern Cooperative Oncology Group.

a Mann–Whitney *U* test.

b Kruskal–Wallis test.

The median baseline CEA level was 5.45 ng/ml for patients with ypN+ compared with 2.45 ng/ml for patients who presented with a CR. After TNT, preoperative CEA levels remained higher in patients with ypN+ (2.21 ng/ml) than in patients with ypT+ ypN0 (1.92 ng/ml) or CR (1.6 ng/ml), whereas post-operative CEA distribution was not associated with pathological outcome (Supplementary Figure S1, available at https://doi.org/10.1016/j.esmogo.2026.100355). The probability of CR was 35.83% in patients with a baseline CEA level <3 ng/ml, compared with 11.32% in patients with CEA levels ≥10 ng/ml. CEA delta (CEA post-TNT minus baseline CEA) was not associated with the probability of CR (Supplementary Figure S2, available at https://doi.org/10.1016/j.esmogo.2026.100355).

The proportion of patients with CR decreased from 50% in cT2 patients (5 of 10) to 26% in cT3 patients (65 of 251) and to 11% in cT4 patients (5 of 45; chi-square test, *P* = 0.048). There was no significant association between CR rate and cN0 stage [CR rate: cN0, 27% (8 of 30) versus cN+, 25% (66 of 269)] or tumor localization [CR rate: low, 25.6% (33 of 129), intermediate, 25.3% (37 of 146), and high, 12.5% (3 of 24)]. Using an Akaike information criterion model selection method considering all parameters listed in the methods section, we distinguished between a set of possible models and identified a best-fitting generalized linear model to predict CR, consisting of CEA [estimate −0.05, 95% confidence interval (CI) −0.11 to −0.01; *P* = 0.02], age (estimate 0.03, 95% CI 0.00-0.065, *P* = 0.05), hemoglobin levels (estimate 0.20, 95% CI −0.02 to 0.42, *P* = 0.08), leukocyte counts (estimate −0.187, 95% CI −0.33 to −0.01, *P* = 0.04), and creatinine levels (estimate −1.49, 95% CI −3.43 to 0.44, *P* = 0.13), with an area under the curve (AUC) of 0.725 (95% CI 0.652-0.798). This model exhibited a sensitivity of 0.79 and a specificity of 0.53, associated with a false negative rate of 11.76% and a true positive rate of 36.8%. We carried out an internal validation via bootstrap to estimate the degree of optimism of the model. The adjusted optimism-adjusted AUC was 0.696. Next, we carried out an additional internal validation by randomly splitting the cohort into fitting (75%) and validation (25%) portions. While the median AUC was 0.728 in the training portion, it was 0.687 in the validation portion (Supplementary Figures S3 and S4, available at https://doi.org/10.1016/j.esmogo.2026.100355).

**Figure 2:**
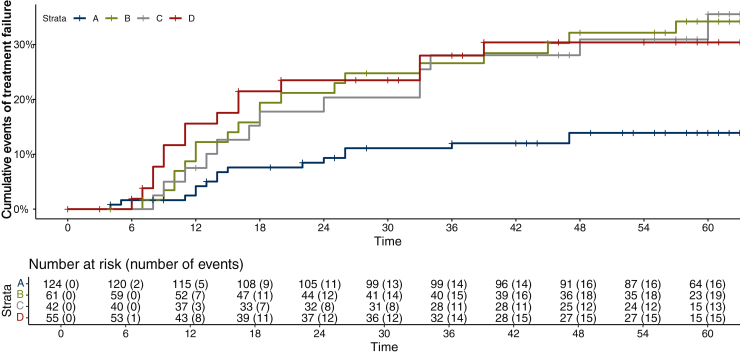
**Cumulative events of TF stratified according to baseline CEA levels (A: CEA <3 ng/ml; B: CEA ≥3 ng/ml but <5 ng/ml; C: CEA ≥5 ng/ml but <10 ng/ml; D: CEA ≥10 ng/ml).** CEA, carcinoembryonic antigen; TF, treatment failure.

In the CAO/ARO/AIO-12 trial, baseline continuous CEA levels were significantly associated with the risk of TF [hazard ratio (HR) 1.01, 95% CI 1.005-1.022, *P* = 0.001] estimated using Fine–Gray regression with death considered a competing risk. Cumulative TF events were stratified according to baseline CEA levels/strata (A: CEA <3 ng/ml, B: CEA ≥3 ng/ml but <5 ng/ml, C: ≥5 ng/ml but <10 ng/ml; D: ≥10 ng/ml). Compared with baseline CEA levels <3 ng/ml, CEA levels between 3 and 5 ng/ml (HR 2.69, 95% CI 1.40-5.19, *P* = 0.003), CEA levels between 5 and 10 ng/ml (HR 2.68, 95% CI 1.31-5.48, *P* = 0.007), and CEA levels >10 ng/ml (HR 2.43, 95% CI 1.19-4.96, *P* = 0.015) were associated with a significantly higher risk of TF (Figure 2).

After a median follow-up period of 60 months (interquartile range 58-62), seven cases of isolated local recurrence (LR) occurred without any simultaneous distant metastasis (DM). Fifty-three cases of DM occurred as isolated progression, and 11 cases of combined DM and LR events occurred. An interval of 3 months before and 1 month after the TF event was accepted for CEA measurement, as defined by electronic case report form entry, to ensure that CEA levels were associated with the event. CEA levels were available for 52 of 71 TF events (73%; Supplementary Figure S5, available at https://doi.org/10.1016/j.esmogo.2026.100355). The median CEA level was 3.5 ng/ml at the time of DM, as 18 patients (43% of patients with CEA available) had a confirmed CEA level >5 ng/ml. The median CEA level was 3.3 ng/ml in patients with isolated LR, with no patients having a CEA elevation >5 ng/ml. The median CEA level was 6.5 ng/ml in patients simultaneously having LR and DM. In patients with initial liver metastases only, the median CEA level at diagnosis was 5.8 ng/ml, compared with 2.6 ng/ml in patients with lung metastases only and 8.3 ng/ml in patients with simultaneous lung and liver metastases.

**Figure 3:**
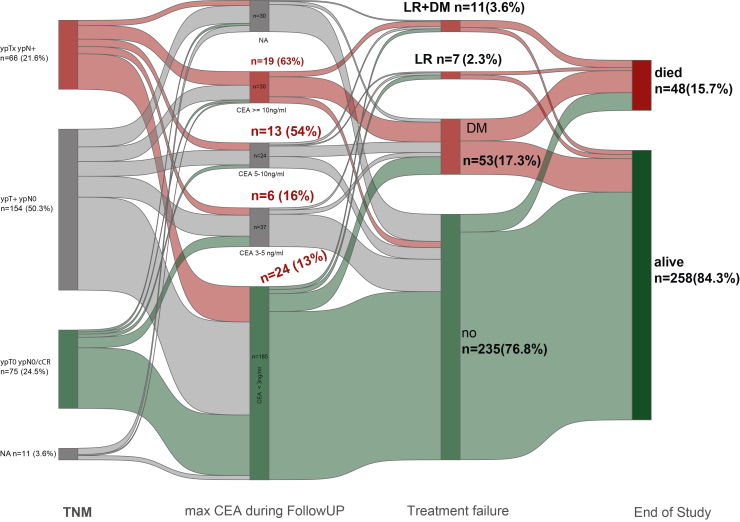
**Sankey diagram of CEA distribution during surveillance and its association with occurrence of TF and death.** CEA, carcinoembryonic antigen; DM, distant metastasis; LR, local recurrence; TF, treatment failure.

A second Sankey diagram was designed to illustrate CEA distribution during surveillance based on pathological outcomes after TNT and their association with TF and OS. The risk of TF was 13% in patients without any CEA elevation >3 ng/ml during the surveillance period, compared with 16% in patients with CEA elevation ≥3 ng/ml but <5 ng/ml, 54% in patients with CEA elevation ≥5 ng/ml but <10 ng/ml, and 63% in patients with CEA elevation ≥10 ng/ml. By the end of the surveillance period, 258 patients (84%) were still alive (Figure 3).

We also investigated how often patients with a baseline CEA level <3 ng/ml developed an increase in CEA level ≥3 ng/ml during the surveillance period (‘CEA-turn’). We identified six patients with CEA elevation after baseline CEA <3 ng/ml. Of these six patients, four (67%) experienced TF associated with the new CEA elevation.

To address the debate surrounding the identification of patients who would benefit from TNT, we carried out multivariate Fine–Gray regression analyses of the risk of TF, using data from the CAO/ARO/AIO-04 trial to enable a comparable analysis. Of 1405 patients in the CAO/ARO/AIO-04 and CAO/ARO/AIO-12 trials, baseline CEA levels could be assessed. Besides baseline cT category and treatment approach, CEA levels remained significantly associated with the risk of TF. Next, we carried out multivariate competing risk regressions in CEA-defined subgroups of patients. Only in the subgroup of patients with baseline CEA levels >10 ng/ml was TNT significantly associated with a lower risk of TF than 5-FU CRT followed by adjuvant 5-FU (Supplementary Figure S6, available at https://doi.org/10.1016/j.esmogo.2026.100355). Interestingly, ECOG performance status (HR 1.63, 95% CI 1.01-2.62, *P* = 0.044) was the only other factor significantly associated with the risk of TF in this subgroup, rather than cT category, cN category, tumor localization, or grading (Table 2).

**Table 2:** Multivariate analyses of treatment failure stratified by pretreatment CEA levels

Parameter	*n*	TF HR (95% CI) ALL	*P*-value	Only patients with CEA < 3 ng/ml	*P*-value	Only patients with CEA 3-5 ng/ml	*P*-value	Only patients with CEA 5-10 ng /ml	*P*-value	Only patients with CEA ≥ 10 ng/ml	*P*-value
CEA (ng/ml)											
<3	680	1									
3-5	225	1.82 (1.30-2.56)	**<0.001**								
5-10	231	2.00 (1.44-2.78)	**<0.001**								
>10	269	2.57 (1.91-3.46)	**<0.001**								
Sex											
Male	984	1		1		1		1		1	
Female	421	0.97 (0.75-1.26)	0.8	0.78 (0.49-1.24)	0.3	0.89 (0.55-1.44)	0.6	1.21 (0.67-2.2)	0.5	1.17 (0.73-1.85)	0.5
Age	1405	0.99 (0.98-1.00)	0.2	0.99 (0.96-1.01)	0.2	1.01 (0.99-1.03)	0.2	1 (0.97-1.02)	0.7	0.98 (0.96-1.0)	0.1
Treatment											
5-FU	565	1		1		1		1		1	
5-FU/Ox	558	0.73 (0.56-0.94)	**0.016**	0.78 (0.5-1.21)	0.3	0.94 (0.59-1.49)	0.8	0.58 (0.31-1.06)	0.074	0.73 (0.45-1.19)	0.2
TNT	282	0.64 (0.47-0.89)	**0.008**	0.63 (0.34-1.18)	0.2	0.74 (0.45-1.21)	0.2	0.99 (0.5-1.95)	0.9	0.45 (0.23-0.86)	**0.017**
ECOG											
0	1086	1		1		1		1		1	
1/2	300	1.31 (0.99-1.73)	0.06	1.39 (0.84-2.33)	0.2	0.82 (0.46-1.45)	0.5	1.28 (0.67-2.44)	0.5	1.63 (1.01-2.62)	**0.044**
cT category											
2	60	1		1		1		1		1	
3	1219	2.61 (1.04-6.54)	**0.04**	1.12 (0.4-3.12)	0.8	27.3 (13.2-56.8)	**<0.001**	28.9 (13.6-61.0)	**<0.001**	2.85 (0.32-25.6)	0.4
4	121	3.14 (1.19-8.32)	**0.02**	1.51 (0.42-5.49)	0.5	24.5 (9.6-62.8)	**<0.001**	30.3 (9.8-93.7)	**<0.001**	3.91 (0.42-36.4)	0.2
cN category											
0	296	1		1		1		1		1	
1	1075	1.01 (0.76-1.34)	0.9	0.89 (0.55-1.42)	0.6	2.17 (1.15-4.08)	**0.016**	1.13 (0.61-2.1)	0.7	0.69 (0.41-1.18)	0.2
Tumor localization											
Low	537	1		1		1		1		1	
Intermediate	713	0.85 (0.66-1.1)	0.2	0.89 (0.57-1.38)	0.6	0.66 (0.44-1.0)	0.05	1 (0.58-1.74)	0.9	0.85 (0.52-1.39)	0.5
High	135	0.92 (0.61-1.4)	0.7	0.92 (0.45-1.92)	0.8	0.55 (0.22-1.35)	0.2	0.78 (0.3-2.03)	0.6	1.31 (0.62-2.77)	0.5
Grading											
G1	75	1		1		1		1		1	
G2	1127	0.97 (0.56-1.68)	0.9	0.87 (0.36-2.21)	0.8	1.89 (0.67-5.44)	0.2	0.43 (0.13-1.44)	0.2	1.09 (0.38-3.17)	0.9
G3	109	1.65 (0.88-3.1)	0.12	2.03 (0.76-5.4)	0.2	3.15 (0.87-11.4)	0.08	0.61 (0.14-2.62)	0.5	1.54 (0.44-5.41)	0.5

HR for TF calculated with death as competing risk. Bolded *P* values are statistically significant.

5-FU, 5-fluorouracil; CEA, carcinoembryonic antigen; CI, confidence interval; ECOG, Eastern Cooperative Oncology Group; HR, hazard ratio; Ox, oxaliplatin; TF, treatment failure.

## Discussion

This study emphasizes both the importance and the limitations of CEA measurement in the initial risk stratification of patients with locally advanced rectal cancer and its role in surveillance strategies. However, its poor association with LR may limit its utility in watch-and-wait protocols.

The RAPIDO trial (short-course radiotherapy followed by FOLFOX) and the PRODIGE23 trial (induction folfirinox followed by CRT) established the TNT treatment protocol for locally advanced rectal cancer, as they reported improved disease-free survival (DFS), attributed to a reduction in the rate of DM, and an OS benefit compared with standard CRT with or without adjuvant CT.^1^^,^^17^ Initially, the RAPIDO trial group did not report whether baseline CEA levels predicted benefit from TNT, but in a *post hoc* analysis, they confirmed that low pretreatment CEA levels (<5 ng/ml) were associated with a higher chance of CR.^10^

In the PRODIGE23 trial, however, the forest plot implied that CEA groups split by 5 ng/ml showed no significant interaction in terms of different treatment effects; therefore, the TNT protocol seems to be beneficial in both groups. Interestingly, the relative number of patients experiencing events (treatment failures) was higher in the subgroup with baseline CEA levels ≤5 ng/ml (25.6% compared with 23.6% in the experimental arm and 36.9% compared with 32.3% in the standard arm).^18^ These data indicate that established cut-off levels for CEA may be inaccurate. Notably, our study found an increased risk of TF in patients with a baseline CEA level ≥3 ng/ml compared with those with a level <3 ng/ml. However, even though CEA levels investigated as a continuous parameter indicated an association between absolute level and risk of TF, this did not lead to difference in HRs between subgroups stratified by baseline levels of CEA.

In both the PROSPECT and FOWARC trials, which challenged the role of radiotherapy as part of the neoadjuvant treatment regime for locally advanced rectal cancer, no subgroup analysis based on CEA levels was reported. CEA levels showed no significant association with 3-year DFS, and the authors argued that inconsistency of CEA cut-offs may at least partly explain the equivocal findings in the literature.^19^^,^^20^

Even considering the limitations of using an unmatched cohort from another trial with a different recruitment period, it should be noted that we observed a significant benefit of intensified treatment in the group with higher baseline CEA levels, but not in the other subgroups, even when baseline factors such as cT and cN category were considered. Although modern staging modalities obviously include circumferential resection margin, extramural venous invasion, and cT3 category subclassification (cT3a-b versus cT3c-d), it may be questionable whether high magnetic resonance imaging (MRI) quality can be consistently achieved worldwide. Therefore, CEA measurements should be considered not only as a standard parameter of initial staging procedures, but also in the risk stratification of rectal cancer patients, to address the risk of overtreatment.^21^ It may be worth noting that, in our study, cT category was significantly associated with the probability of CR. However, maybe due to the small number of cT2 (*n* = 10) and cT4 (*n* = 45) tumors relative to cT3 (*n* = 251) tumors, cT category was not selected as a parameter in the best-fitted generalized prediction model, in contrast to baseline CEA levels.

The established surveillance strategies for rectal cancer after surgery are based on serial CEA measurements, computed tomography scans of the thorax and abdomen, history and physical examination, and colonoscopy. In the case of organ preservation approaches, a more intensive follow-up schedule is recommended.^5^^,^^6^ However, even when considering the difference in risk of TF, guidelines do not recommend an intensified surveillance protocol for ‘high-risk’ patients (e.g. ypT4, ypN+).^22^

Shen et al.^11^ showed that patients with normal baseline CEA levels can experience CEA elevation during follow-up (CEA-turn), which can be associated with TF. Although CEA surveillance in patients with normal baseline CEA levels may not be highly sensitive, the potential risk of overlooking patients with CEA elevation, which is potentially associated with a worse prognosis, should be avoided. Therefore, CEA levels should also be monitored in patients with normal baseline levels. However, this study strengthens the conclusion of Nicholson et al.^23^ that CEA measurement alone is not sensitive enough to be used as the only surveillance strategy. It should be noted that, in our study, the risk of TF was only 13% in patients who had no CEA elevation ≥3 ng/ml during surveillance, compared with 54% and 63% in patients who did report such elevation ≥5 ng/ml or ≥10 ng/ml, respectively.

Although performing adequate surveillance for patients with rectal cancer is beyond question, the potential benefits of intensive surveillance protocols are not. Although a meta-analysis by Pita-Fernández et al.^24^ reported an OS benefit due to intensified surveillance strategies and an increased probability of curative surgery in colorectal cancer patients after surgery, the PRODIGE13 trial, while reporting an increase in curative-intent salvage surgery, did not find a survival benefit.^25^ Further integration of novel techniques, such as circulating tumor DNA (ctDNA), has so far failed to demonstrate clinical benefits in the surveillance of colorectal cancer patients.^26^ However, some progress has been made in predicting pCR in rectal cancer using serial ctDNA samples. Furthermore, analyzing median variant allele frequency and tumor mutational burden rate of ctDNA samples could further improve the use of serial ctDNA measurements for predicting response and prognosis.^27^ However, most studies lack prospective external validation, which is crucial for broader clinical implications.^28^ Although the evidence is still in its preliminary stages, a small study by Murahashi et al.^29^ (*n* = 85) found that combining post-operative CEA and ctDNA levels in rectal cancer patients may provide a robust prediction of recurrence.

While acknowledging that an optimal strategy has yet to be established, most guidelines from the European Society for Medical Oncology, American Society of Clinical Oncology, and National Comprehensive Cancer Network recommend regular CEA measurements and computed tomography scans of the abdomen and chest during the surveillance period.^5^^,^^6^^,^^30^ Notably, German guidelines explicitly do not recommend computed tomography scans, preferring ultrasound and X-ray, as no superiority of computed tomography scans has been reported, and therefore recommending the most cost-effective techniques.^31^ As postsurgical status is highly associated with the risk of TF in rectal cancer, it may be more appropriate to investigate the benefits of intensified surveillance strategies only in these high-risk subgroups of patients.^22^ Nevertheless, as no isolated LR was associated with substantial CEA elevation, it is questionable whether CEA measurements may be sufficient to substitute costly MRI and rectoscopy in patients undergoing a watch-and-wait approach with the aim of organ preservation. Renehan et al.^32^ proposed in their meta-analysis that a surveillance protocol itself due to regular medical visits may decrease noncancer mortality or even improve psychological well-being in patients, as data on the impact of surveillance strategies on quality of life are underrepresented, as it should be obvious that surveillance strategies should only be recommended to patients who are capable and willing to undergo salvage treatment, if necessary.

Our study has several limitations. Firstly, this was a *post hoc* analysis, and no adjustment for multiple testing was implemented. Secondly, rather than performing a pair-match analysis within the CEA subgroup analysis, we included relevant baseline parameters in the multivariate regression models. Other differences between the CAO/ARO/AIO-04 and CAO/ARO/AIO-12 trials also need to be considered, such as differences in inclusion criteria, staging modalities, and the period over which recruitment took place. Thirdly, smoking status was not assessed in the trial cohort. CEA levels are elevated in smokers due to an inflammatory reaction.^33^ As the amount and duration of smoking may be associated with the degree of CEA elevation, it may be recommended that smoking status is questioned not only in terms of binary results.^34^ Fourthly, as CEA levels are associated with sex, age, and BMI, and are at least affected by liver or kidney function, modified CEA levels should improve sensitivity. It was beyond the scope of this study to propose and test a formula for a modified CEA score that could improve sensitivity compared with standard CEA cut-off levels, as the necessary data were not collected during the follow-up period. Fifthly, we acknowledge that our generalized linear regression shows some indication of overfitting in the internal validation approach and that no external validation cohort comparison was carried out. Moreover, it may be considered worthwhile to incorporate alternative prediction models, such as spline regression models, in subsequent studies based on larger datasets. Sixthly, we acknowledge that limitation in interlaboratory reproducibility over the recruitment period of these two trials should be considered.

This study validated the role of CEA in surveillance strategies for curative treatment of rectal cancer. Furthermore, we provide evidence that CEA levels could be useful in assessing the likelihood of a complete response after TNT, given that CEA measurements are widely available and cost-effective. In our study, however, the limited sensitivity of CEA in cases of isolated locoregional recurrence raises questions about the appropriateness of repeated CEA measurements during surveillance as a potential substitute for costly and personal intensive radiological or endoscopic surveillance protocols in patients undergoing an organ preservation approach.

## Supplementary data

Supplementary Figure S6

Supplementary Table S1 Correlation of BMI and laboratory parameters with baseline CEA levels
