# Supplementary material for: Reassessing the role of CEA in the treatment of rectal cancer in the era of TNT
Source: ESMO Gastrointest Oncol. 2026 Jun 29;13(Pt B):100355. doi: 10.1016/j.esmogo.2026.100355 (PMC13459590; doi:10.1016/j.esmogo.2026.100355)
Supplement: Supplementary file 1 [file mmc1.pdf]

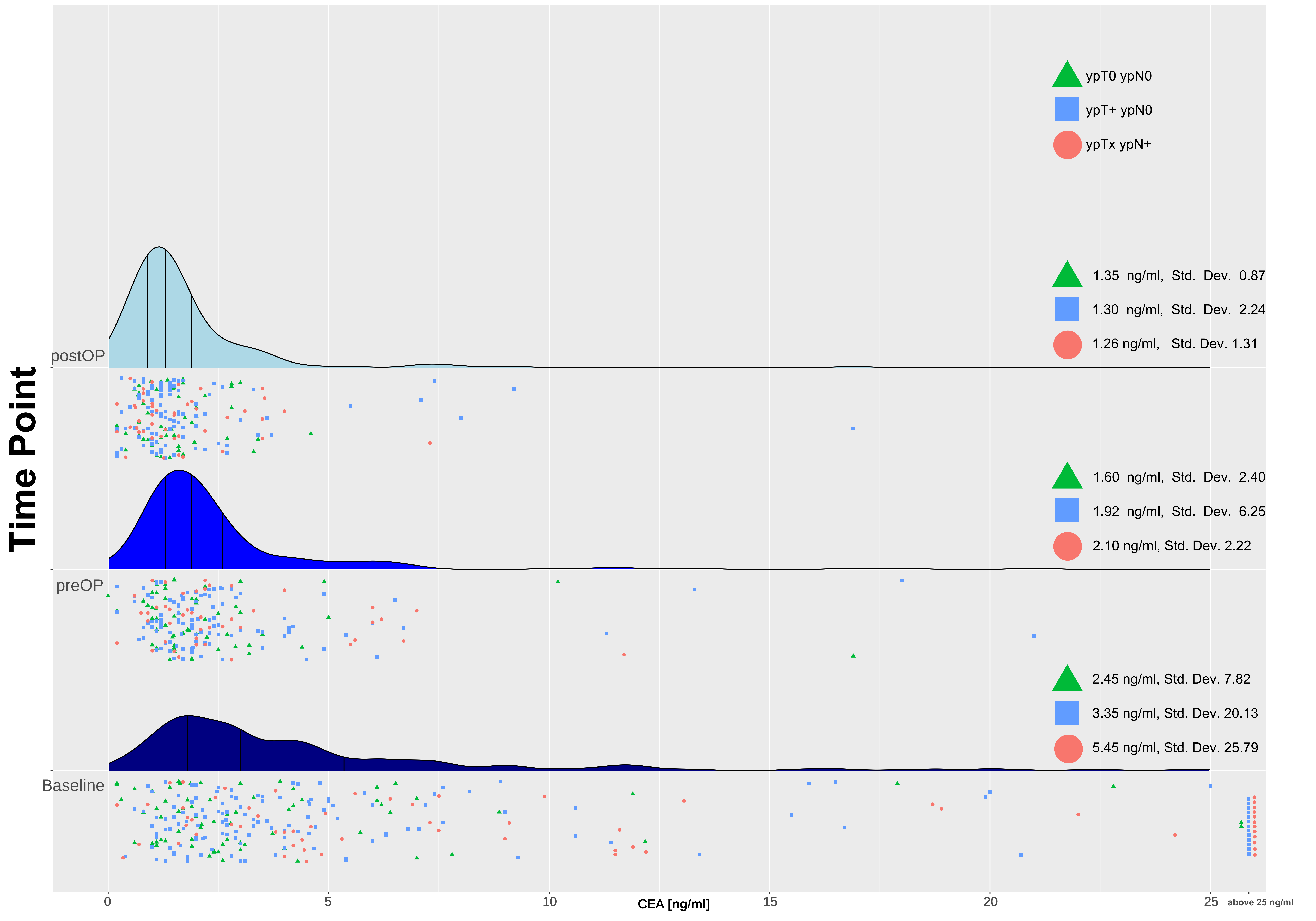

Time Point
postOP
preOP
Baseline
ypT0 ypN0
ypT+ ypN0
ypTx ypN+
1.35 ng/ml, Std. Dev. 0.87
1.30 ng/ml, Std. Dev. 2.24
1.26 ng/ml, Std. Dev. 1.31
1.60 ng/ml, Std. Dev. 2.40
1.92 ng/ml, Std. Dev. 6.25
2.10 ng/ml, Std. Dev. 2.22
2.45 ng/ml, Std. Dev. 7.82
3.35 ng/ml, Std. Dev. 20.13
5.45 ng/ml, Std. Dev. 25.79
0
5
10
15
20
25
above 25 ng/ml
CEA [ng/ml]
