# Supplementary material for: Reassessing the role of CEA in the treatment of rectal cancer in the era of TNT
Source: ESMO Gastrointest Oncol. 2026 Jun 29;13(Pt B):100355. doi: 10.1016/j.esmogo.2026.100355 (PMC13459590; doi:10.1016/j.esmogo.2026.100355)
Supplement: Supplementary file 2 [file mmc2.pdf]

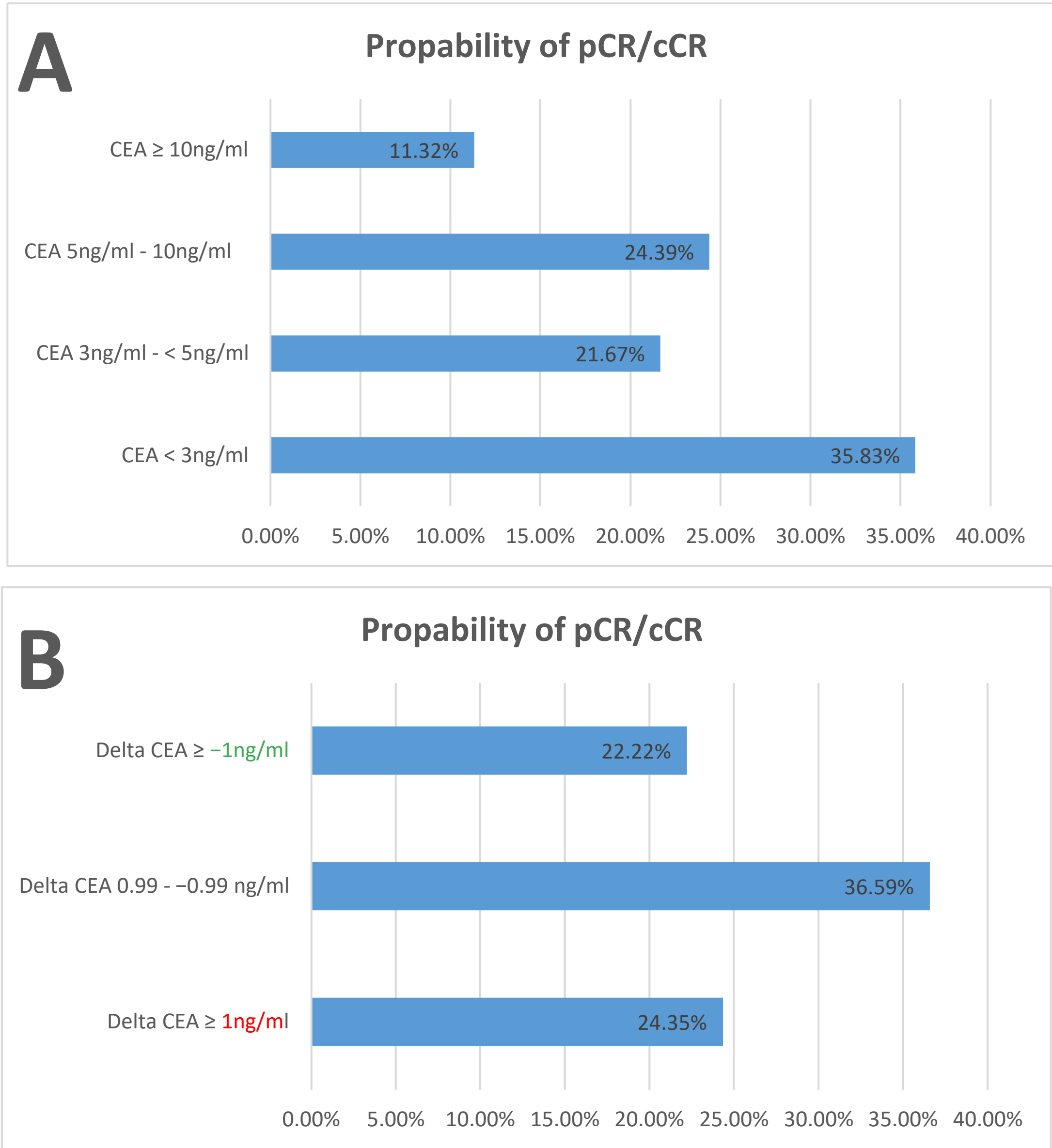
A
Propability of pCR/cCR
CEA ≥ 10ng/ml
11.32%
CEA 5ng/ml - 10ng/ml
24.39%
CEA 3ng/ml - < 5ng/ml
21.67%
CEA < 3ng/ml
35.83%
0.00% 5.00% 10.00% 15.00% 20.00% 25.00% 30.00% 35.00% 40.00%
B
Propability of pCR/cCR
Delta CEA ≥ −1ng/ml
22.22%
Delta CEA 0.99 - −0.99 ng/ml
36.59%
Delta CEA ≥ 1ng/ml
24.35%
0.00% 5.00% 10.00% 15.00% 20.00% 25.00% 30.00% 35.00% 40.00%
