# Supplementary material for: Reassessing the role of CEA in the treatment of rectal cancer in the era of TNT
Source: ESMO Gastrointest Oncol. 2026 Jun 29;13(Pt B):100355. doi: 10.1016/j.esmogo.2026.100355 (PMC13459590; doi:10.1016/j.esmogo.2026.100355)
Supplement: Supplementary file 3 [file mmc3.pdf]

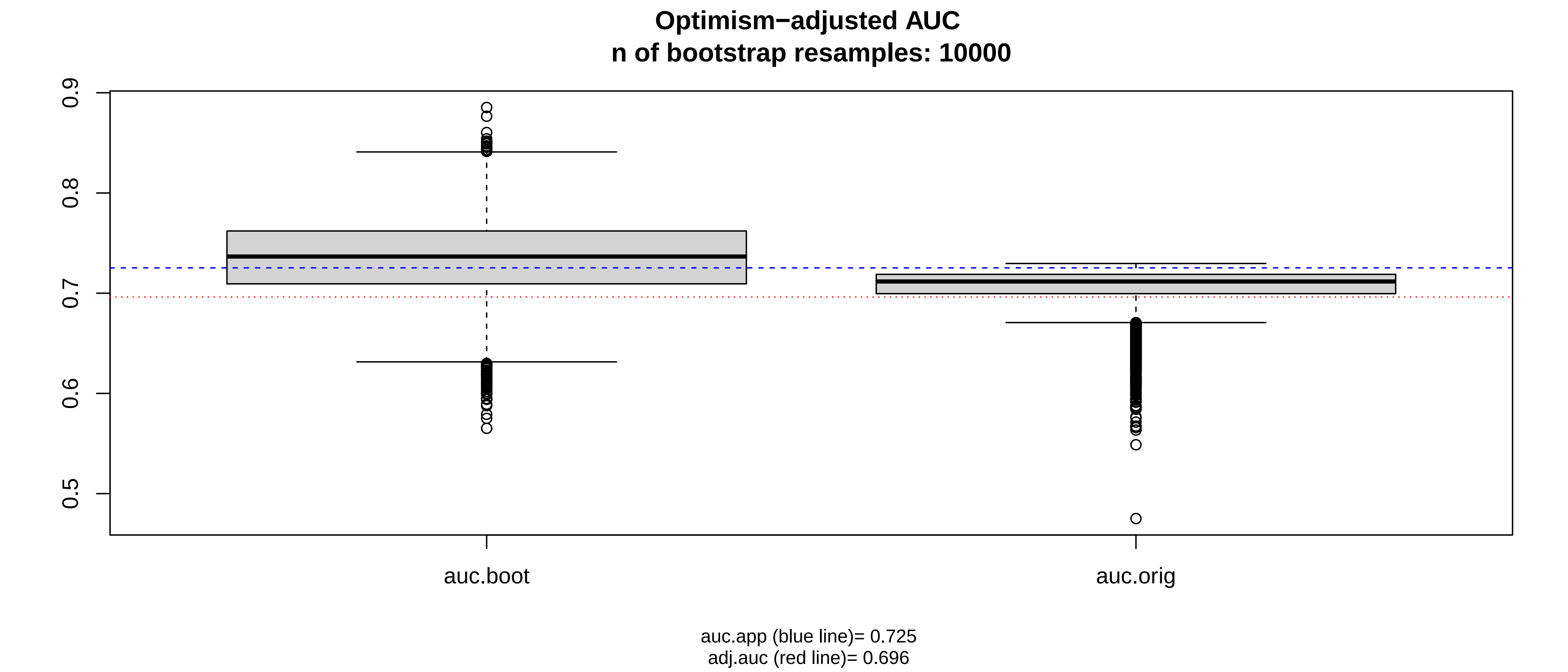
Optimism−adjusted AUC
n of bootstrap resamples: 10000
0.9
0.8
0.7
0.6
0.5
auc.boot
auc.orig
auc.app (blue line)= 0.725
adj.auc (red line)= 0.696
