# Supplementary material for: Reassessing the role of CEA in the treatment of rectal cancer in the era of TNT
Source: ESMO Gastrointest Oncol. 2026 Jun 29;13(Pt B):100355. doi: 10.1016/j.esmogo.2026.100355 (PMC13459590; doi:10.1016/j.esmogo.2026.100355)
Supplement: Supplementary file 4 [file mmc4.pdf]

Cross−validated AUC
n of iterations: 10000

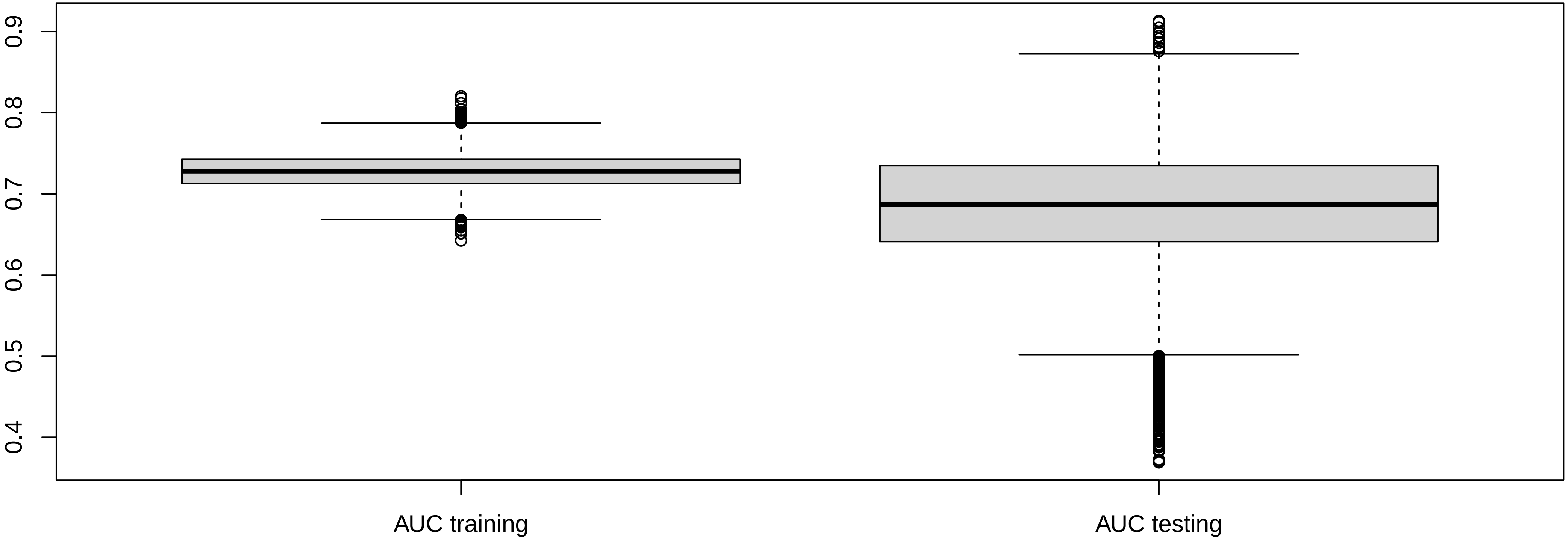

AUC full sample= 0.725
AUC training min, median, max: 0.642 0.728 0.105
AUC testing min, median, max: 0.369 0.687 0.913
