# Supplementary material for: Reassessing the role of CEA in the treatment of rectal cancer in the era of TNT
Source: ESMO Gastrointest Oncol. 2026 Jun 29;13(Pt B):100355. doi: 10.1016/j.esmogo.2026.100355 (PMC13459590; doi:10.1016/j.esmogo.2026.100355)
Supplement: Supplementary file 5 [file mmc5.pdf]

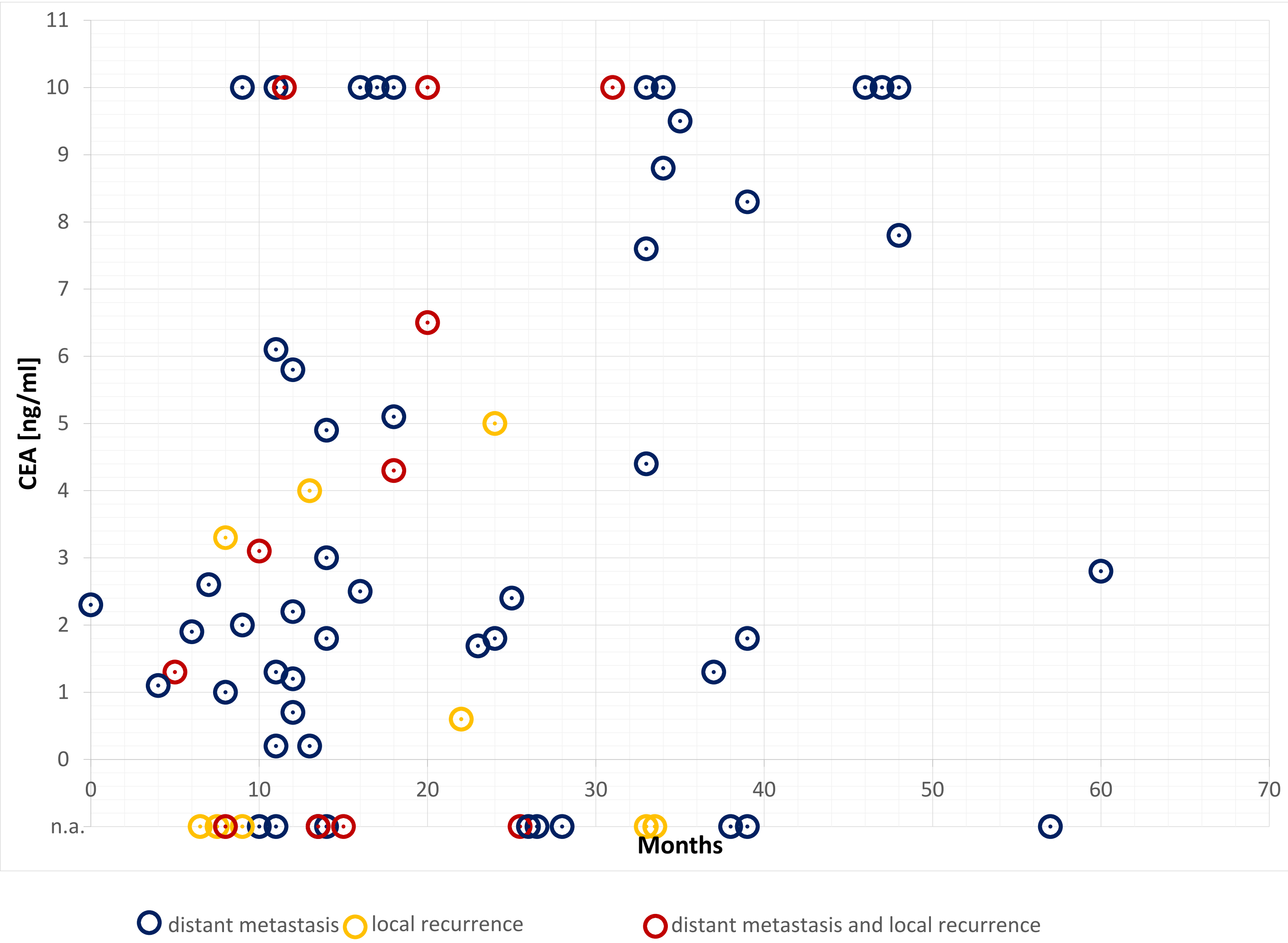
CEA [ng/ml]
Months
n.a.
0
1
2
3
4
5
6
7
8
9
10
11
0
10
20
30
40
50
60
70
distant metastasis
local recurrence
distant metastasis and local recurrence
