# Supplementary material for: Reassessing the role of CEA in the treatment of rectal cancer in the era of TNT
Source: ESMO Gastrointest Oncol. 2026 Jun 29;13(Pt B):100355. doi: 10.1016/j.esmogo.2026.100355 (PMC13459590; doi:10.1016/j.esmogo.2026.100355)
Supplement: Supplementary file 6 [file mmc6.pdf]

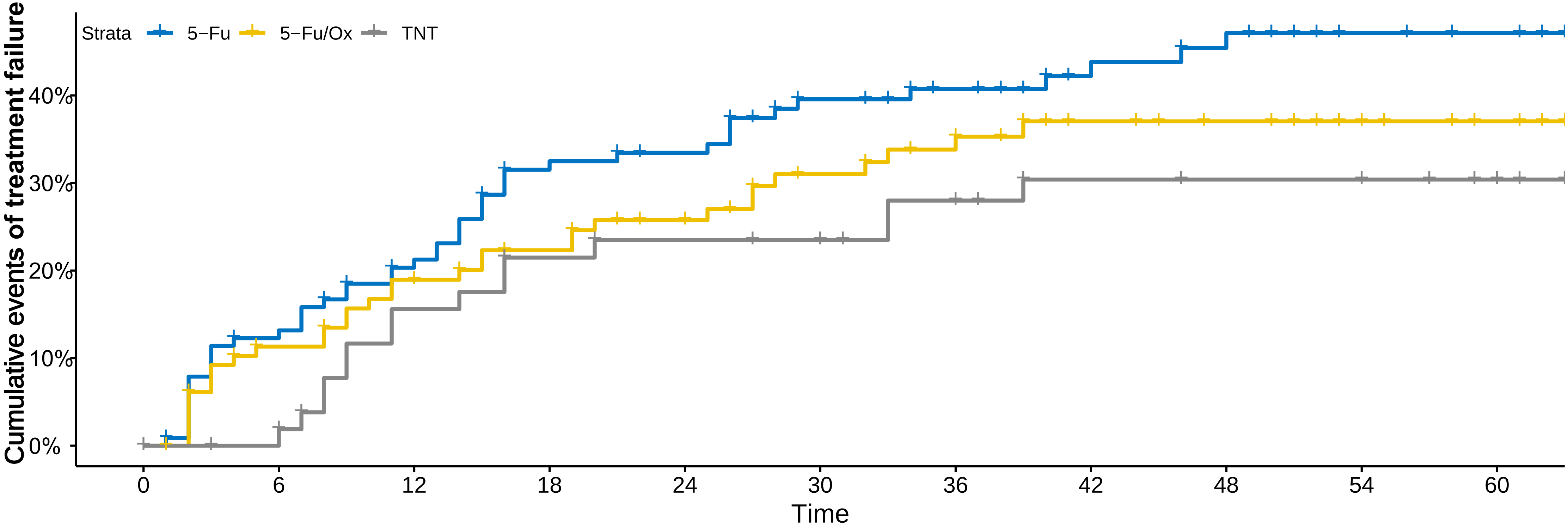

Number at risk (number of events)

Strata	0	6	12	18	24	30	36	42	48	54	60
5−Fu	115 (0)	99 (15)	86 (24)	71 (36)	67 (37)	54 (43)	48 (44)	36 (46)	32 (48)	18 (48)	16 (48)
5−Fu/Ox	99 (0)	82 (11)	74 (18)	68 (21)	60 (24)	50 (28)	45 (31)	29 (32)	25 (32)	18 (32)	14 (32)
TNT	55 (0)	53 (1)	43 (8)	39 (11)	37 (12)	36 (12)	32 (14)	28 (15)	27 (15)	27 (15)	15 (15)

Time

Cumulative number of censoring

Strata	0	6	12	18	24	30	36	42	48	54	60
5−Fu	0	2	6	9	11	18	23	34	36	49	51
5−Fu/Ox	0	6	8	10	17	21	25	38	42	50	53
TNT	1	3	4	5	6	8	10	12	13	15	30

Time
