# Supplementary material for: Reassessing the role of CEA in the treatment of rectal cancer in the era of TNT
Source: ESMO Gastrointest Oncol. 2026 Jun 29;13(Pt B):100355. doi: 10.1016/j.esmogo.2026.100355 (PMC13459590; doi:10.1016/j.esmogo.2026.100355)
Supplement: Supplementary file 7 [file mmc7.docx]

| Parameter | Number | Correlation Coefficient | P value |
| --- | --- | --- | --- |
| BMI [kg/m²] | 279 | -0.002 | 0.97* |
| Hb [g/dl] | 281 | -0.048 | 0.43* |
| Leukocytes [/nl] | 281 | 0.0975 | 0.10* |
| Platelets [/nl] | 281 | 0.197 | **<0.001*** |
| Na [mmol/l] | 281 | 0.006 | 0.92* |
| K [mmol/l] | 282 | 0.034 | 0.57* |
| Ca [mmol/l] | 267 | 0.021 | 0.73* |
| Krea [mg/dl] | 281 | -0.151 | **0.01*** |
| Bili [mg/dl] | 276 | -0.220 | **<0.001*** |
| GOT [U/l] | 268 | 0.003 | 0.97* |
| GPT [U/l] | 278 | -0.037 | 0.66* |
| LDH [U/l] | 261 | 0.031 | 0.62* |
| AP [U/l] | 276 | 0.130 | **0.03*** |
| Protein [g/dl] | 255 | 0.038 | 0.55* |
| Alb [g/dl] | 261 | -0.055 | 0.38* |

*Spearman Correlation
